# *In-silico* prediction and deep-DNA sequencing validation indicate phase variation in 115 *Neisseria meningitidis* genes

**DOI:** 10.1186/s12864-016-3185-1

**Published:** 2016-10-28

**Authors:** Emilio Siena, Romina D’Aurizio, David Riley, Hervé Tettelin, Silvia Guidotti, Giulia Torricelli, E. Richard Moxon, Duccio Medini

**Affiliations:** 1 GSK Vaccines, 53100 Siena, Italy; 2 Institute for Genome Sciences, University of Maryland School of Medicine, Baltimore, MD 21201 USA; 3 Medical Sciences Division, Weatherall Institute of Molecular Medicine, John Radcliffe Hospital, Oxford, OX3 9DS UK; 4 Present address: Institute of Informatics and Telematics and Institute of Clinical Physiology, National Research Council, 56124 Pisa, Italy; 5 Present address: Personal Genome Disgnostics inc., Baltimore, MD 21224 USA

**Keywords:** *Neisseria meningitidis*, Phase variation, Contingency loci, Comparative genomics, Host-pathogen interaction

## Abstract

**Background:**

The *Neisseria meningitidis* (*Nm*) chromosome shows a high abundance of simple sequence DNA repeats (SSRs) that undergo stochastic, reversible mutations at high frequency. This mechanism is reflected in an extensive phenotypic diversity that facilitates *Nm* adaptation to dynamic environmental changes. To date, phase-variable phenotypes mediated by SSRs variation have been experimentally confirmed for 26 *Nm* genes.

**Results:**

Here we present a population-scale comparative genomic analysis that identified 277 genes and classified them into 52 strong, 60 moderate and 165 weak candidates for phase variation. Deep-coverage DNA sequencing of single colonies grown overnight under non-selective conditions confirmed the presence of high-frequency, stochastic variation in 115 of them, providing circumstantial evidence for their phase variability.

We confirmed previous observations of a predominance of variable SSRs within genes for components located on the cell surface or DNA metabolism. However, in addition we identified an unexpectedly broad spectrum of other metabolic functions, and most of the variable SSRs were predicted to induce phenotypic changes by modulating gene expression at a transcriptional level or by producing different protein isoforms rather than mediating on/off translational switching through frameshifts.

Investigation of the evolutionary history of SSR contingency loci revealed that these loci were inherited from a *Nm* ancestor, evolved independently within *Nm*, or were acquired by *Nm* through lateral DNA exchange.

**Conclusions:**

Overall, our results have identified a broader and qualitatively different phenotypic diversification of SSRs-mediated stochastic variation than previously documented, including its impact on central *Nm* metabolism.

**Electronic supplementary material:**

The online version of this article (doi:10.1186/s12864-016-3185-1) contains supplementary material, which is available to authorized users.

## Background

*Neisseria meningitidis (Nm)* is a Gram-negative, encapsulated bacterium that is present, as a commensal organism, in the nasopharyngeal cavity of five to ten percent of the adult population [1, 2]. Despite its prevalence as a harmless organism some strains, for reasons not yet completely understood, can cross the epithelial barrier and enter the bloodstream, causing septicemia and life-threatening disease [3–6].

In order to maximize its fitness in the diverse and changing environments offered by the host-pathogen interplay, *Nm* has evolved multiple and complementary adaptive strategies [7, 8]. One such mechanism is represented by Simple Sequence Repeats (SSRs), contiguous iterations of short DNA motifs, generally assumed to range from 1 to 10 nucleotides in length [9].

SSRs, often called contingency loci, are hyper-mutable DNA sequences mediating high frequency, stochastic, reversible and heritable genotypic switching [10], whose number of tandemly repeated motifs can vary with relatively high frequencies [11, 12]. A variable number SSR (VNSSR) located within a coding sequence can change translation by introducing frameshifts in the reading frame [13–15] or, when located in the proximity of a promoter, can modulate transcription either by switching between alternative translational start sites [11, 16] or by altering the promoter sequence [17–19].

The first *Nm* genome-wide SSRs analyses were based on the single genome sequences determined for the MC58 [9] and Z2491 [20] strains. Putative phase-variable genes were predicted on the basis of *cis* factors such as SSR sequence, number of repetitions and sequence context. For a subset of genes phase variation was confirmed experimentally.

Comparative investigations, in which the genomic sequences of strains *N. gonorrhoeae* FA1090 and *Nm* FAM18 were added to the analysis, suggested that the presence of length polymorphisms among orthologous SSRs present in different genomes is a reliable predictor of phase variation [21, 22]. Overall, 78 putative phase variable genes were reported in the *Neisseria* genus, of which 67 were specific to the *Nm* species and included genes involved in cell adhesion (adhesins), capsule formation (evasins), biosynthesis of the lipopolysaccharide layer (e.g. *LgtA* and *lgtE*), cell-surface receptors (e.g. the *HmbR* iron-acquisition receptor) and restriction/modification systems [22]. Among these, phase variation was confirmed experimentally for only 26 genes, primarily combining PCR with immunoblotting techniques [17, 23–32] or with northern blotting and quantitative PCR [13, 17, 33–35].

Here, we report that the limited number of sequences employed in previous VNSSRs studies, together with the lack of high-throughput technologies (next-generation sequencing, NGS) suitable for a large-scale validation of putative phase variable genes, have potentially led to an underestimation of the overall impact of SSR-mediated phase variation on *Nm* phenotype and fitness. Also, the evolutionary dynamics underlying the generation of VNSSRs in *Nm* and other species has not been fully elucidated.

Recently, new insights into the *Nm* population structure and dynamics have been generated through a comparative genomic analysis based on the complete genome sequences of 20 *Nm* strains, including multiple isolates from each of the most virulent clonal complexes [36]. Also, the advent of NGS technologies have allowed for an unprecedented sequencing depth across the whole genome, such that the presence of mixed populations at specific loci of a single genome can be detected with high throughput, including those variants occurring at low frequencies during bacterial replication [37–40].

In the present study we took advantage of *Nm* pan-genome data to map, characterize and infer functional impact and evolutionary properties of SSR contingency loci, and NGS to experimentally validate *in silico* predictions on stochastic genotypic switching.

## Results

### Two hundred seventy-seven meningococcal genes are associated with simple sequence repeats that show inter-strain length polymorphisms

An average of 4243 SSRs were identified in each genome, the majority of which (95 %) were represented by homopolymeric tracts (Additional file 1). The number of SSRs was similar across genomes (coefficient of variation = 3 %) and their abundance was found to be significantly higher than random expectation (*p* = 6.8e-8; Additional file 2: Figure S1). Significant deviation from neutrality was also observed for each individual SSR type.

We identified 6295 clusters of orthologous SSRs, 35 % (2183) of which were represented in every genome analyzed. Among these, 324 clusters had polymorphisms in the repeat tract length across different genomes, located either in intragenic regions (166, 51 %) or in the associated gene’s upstream region (158, 49 %). These were selected for further analyses based on their potential to change gene expression.

Consistent with previous findings [9], homopolymeric tracts were the most abundant simple sequence repeat type, with 288 VNSSRs clusters divided into 229 A/T and 59 G/C tracts. The remaining 36 clusters were represented by tandem repeats of two- to nine-nucleotides motifs (Additional file 3).

Positional analysis identified 166 (51 %) intragenic and 158 (49 %) intergenic VNSSRs, respectively. Interestingly, all VNSSRs with a unit motif of three-, six- and nine-nucleotides, for which a length polymorphism would not result in the disruption of the reading frame, were found to be located within coding sequences (Additional file 3). Finally, thirty-two genes were associated with more than a single VNSSR, resulting in a total of 277 phase variable gene candidates.

### SSR contingency loci show different genotype switching frequencies

**Fig. 1:**
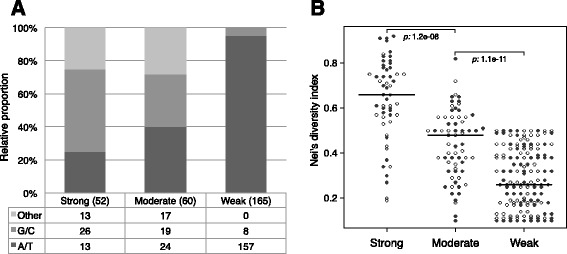
Properties of identified VNSSRs. **a** Relative contribution of different VNSSRs types to the Strong, Moderate and Weak candidate phase variable genes. Other: all non-homopolymeric repeats. G/C: guanine or cytosine variable homopolymeric tracts. A/T: adenine or thymine variable homopolymeric tracts. **b** Nei’s diversity index distribution of VNSSRs associated with the 52 strong, 60 moderate and 165 weak candidate phase variable genes. Genes associated with intragenic and intergenic VNSSRs are represented full and empty circles, respectively. The thick horizontal lines represent distributions median. *p*: Wilcoxon-Mann-Whitney *p* value

Based on repeat variability and intra-strain phylogenetic relationships we stratified the 277 VNSSR-associated genes into 52 strong, 60 moderate and 165 weak candidates for phase variation. Most weak candidate phase variable genes (157, 95 %) were associated with A/T homopolymeric tracts while G/C repeats were the most abundant VNSSR type among the strong candidate genes [26, 50 % (Fig. 1a)]. VNSSRs other than homopolymeric repeats were found in 17 moderate (28 %) and 13 strong (25 %) candidate genes, respectively (Fig. 1a).

Intrinsic variability of VNSSRs clusters, defined as the Nei’s diversity index [41], was then computed for the three gene sets. VNSSRs associated to strong candidate genes were significantly more variable than those associated to moderate genes, which in turn showed higher variability than those linked to weak phase variable gene candidates (Fig. 1b), providing supporting evidence that genes associated with a different likelihood of undergoing phase variation exist and that these can be identified through the analysis of a suitable genome dataset.

A positive correlation between the SSR tract length and repeat instability has been proposed [9, 42], possibly as a result of a decreased proof-reading efficiency of the DNA polymerase over the longer tracts [43]. We could confirm such association for the strong [Pearson correlation coefficient: *R* = 0.6 (homopolymeric tracts) and *R* = 0.73 (non-homopolymeric repeats)] but not for the moderate or weak candidate gene pools (Additional file 2: Figure S2).

### VNSSRs variation is detectable in single colonies grown overnight under non-selective conditions

**Fig. 2:**
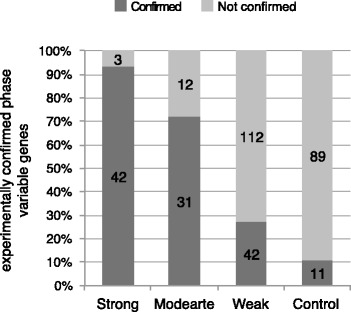
Phase variable genes confirmed by Illumina sequencing. Numbers inside bars indicate the frequency of phase variable genes present in each category. Confirmed: predicted phase variable genes confirmed by deep sequencing data. Not confirmed: predicted phase variable genes not showing evidence of variation. Control: analysis of 100 randomly selected SSRs that were predicted as non-variable by the genomic comparative analysis

The genomes of five of the analyzed strains were re-sequenced at high depth using a next generation sequencing platform with the goal of detecting repeats length polymorphisms occurring in single colonies during an overnight growth. Analysis of the sequencing reads confirmed repeat length polymorphisms in 115 of the 277 predicted phase variable genes, distributed in 42 strong (81 %), 31 moderate (52 %) and 42 weak (25 %) putative phase variable genes (Fig. 2 and Additional file 4). The same procedure applied to 100 randomly chosen, non-variable SSRs detected length polymorphisms in 11 loci (11 %, Fig. 2), a significantly lower proportion compared to VNSSRs (chi-squared test for proportions *p* ≤ 0.01). Additionally, the average frequencies of polymorphic reads (those containing an SSR showing length polymorphism) observed for VNSSRs was 4.5 %, a higher proportion compared to the 11 false positive SSRs (0.7 %) or to the allowed read mapping error rate (≤0.1 %; Phred-scaled MAPping quality ≥30). Frequency distributions of polymorphic reads identified in the 5 genomes are reported in Additional file 2: Figure S3.

Seventy of the 115 validated VNSSRs were present in all five re-sequenced genomes. Among these, 26 (37 %) were shown to be variable in all the 5 genomes analyzed, while 44 (63 %) were confirmed in four or fewer genomes (Additional file 2: Figure S4A). Interestingly, for any one genome there was a strong association between SSR size (number of unit motif repetitions) and the likelihood of finding it variable. Specifically, homopolymeric tracts of 8 or more nucleotides were predominantly found to be variable in four or five genomes (chi-square *p* = 1e-11; Additional file 2: Figure S4B). Because there were only three non-homopolymeric tracts, it was not possible to generalize this conclusion to longer repeats. Additionally, for the 70 experimentally confirmed core VNSSRs, the proportion of polymorphic reads was found to correlate (adjusted *R* ^*2*^ = 0.28; *p* = 1.6e-6; Additional file 2: Figure S5A) with the Nei’s diversity index derived from the comparison of the 20 available genome sequences. Specifically, VNSSRs associated to a Nei’s index >0.5 were mainly characterized by above-average polymorphic reads proportions (chi-square *p* = 0.004; Additional file 2: Figure S5B), meaning that the extent of VNSSRs variability can be predicted with good confidence by the comparison of multiple genomic sequences.

Finally, the number of phase variable genes that would be confirmed by deep sequencing of a wider genome collection was estimated by applying an approach originally proposed for estimating the bacterial pan-genome size [44]. The regression analysis based on the number of validated phase variable genes predicted that if all the 20 genomes were re-sequenced, the number of validated phase-variable genes would have been 146 ± 10 (95 % CI; Additional file 2: Figure S6), while the analysis of 100 genomes could raise the number to 190 ± 23 (95 % CI).

### The pool of genes whose expression is influenced by VNSSRs is wider than previously hypothesized

The pool of putative phase variable genes predicted in this study was compared with the 69 genes previously described or predicted to be phase-variable in *Nm* [22]. Our approach confirmed 24 of the 26 genes whose phase variation had been previously demonstrated in *Nm*, as well as 15 of the 19 putative phase variable genes previously proposed as strong candidates and 8 of the 24 genes previously reported as either moderate or weak candidates (Additional file 2: Figure S7 and Additional file 3). The 22 genes not confirmed by our method, either didn’t satisfy the criteria for SSR search or didn’t show inter-strain length polymorphisms. In the particular case of a poly-C tract located within the NMB1760 coding region, the repeat showed between-strain variation, alternating between the C5 and C6 states. However, since the C5 was below the minimum length cut off (see materials and methods) our approach failed to identify this variable SSR. Remarkably, the wider genome collection allowed for the identification of 230 new putative phase variable genes, predicted as 19 strong, 50 moderate and 161 weak candidates respectively (Additional file 2: Figure S7 and Additional file 3).

### Candidate phase variable genes related to cell surface structure or involved in DNA metabolism are overrepresented

**Fig. 3:**
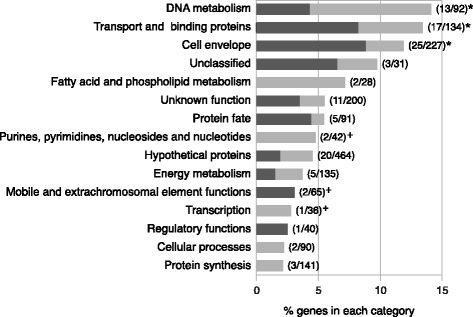
TIGR functional roles represented by the 112 strong and moderate candidate phase variable genes. X-axis represents the proportion of putative phase variable genes present in each annotation. Numbers in brackets represent the number of putative phase variable genes over the total number of genes that are associated to a specific function. Genes associated with intragenic and intergenic VNSSRs are represented in dark and light grey, respectively. ‘*’: Enriched functional roles (Bonferroni adjusted *p* ≤ 0.01). ‘**+**’: Functional roles not previously described to be associated with SSR-mediated regulation in *Nm*

The 112 strong and moderate candidate genes, described in results section 2, distributed across 15 different functional roles [defined as TIGR main roles [45], Fig. 3]. Consistent with previous studies [9, 21] VNSSR-associated genes were found to be primarily involved in host-microbial interplay, with “Cell envelope”, “Transport and binding” and “DNA metabolism” functional roles being significantly overrepresented. Intragenic and intergenic VNSSRs were both present in the enriched categories. Other biological processes included functions related to the peptide synthesis machinery (“Protein synthesis” and “Protein fate”). Additionally, 5 putative phase variable genes were found in functional categories that had not been previously associated to SSR-mediated regulation, such as genes encoding for transposons and prophage related functions, transcription factors and proteins participating in nucleic acids biosynthesis (Fig. 3).

The 165 weak candidate genes were associated with 18 different functional roles, fourteen of which were common to the strong and moderate groups. This subset showed a different profile, whereby genes were significantly overrepresented in the “hypothetical proteins” and “mobile and extrachromosomal element functions” functional annotations (Additional file 2: Figure S8).

### Most VNSSRs are predicted to induce phenotypic changes by modulating the level of gene expression rather than mediating on/off translational switching

**Fig. 4:**
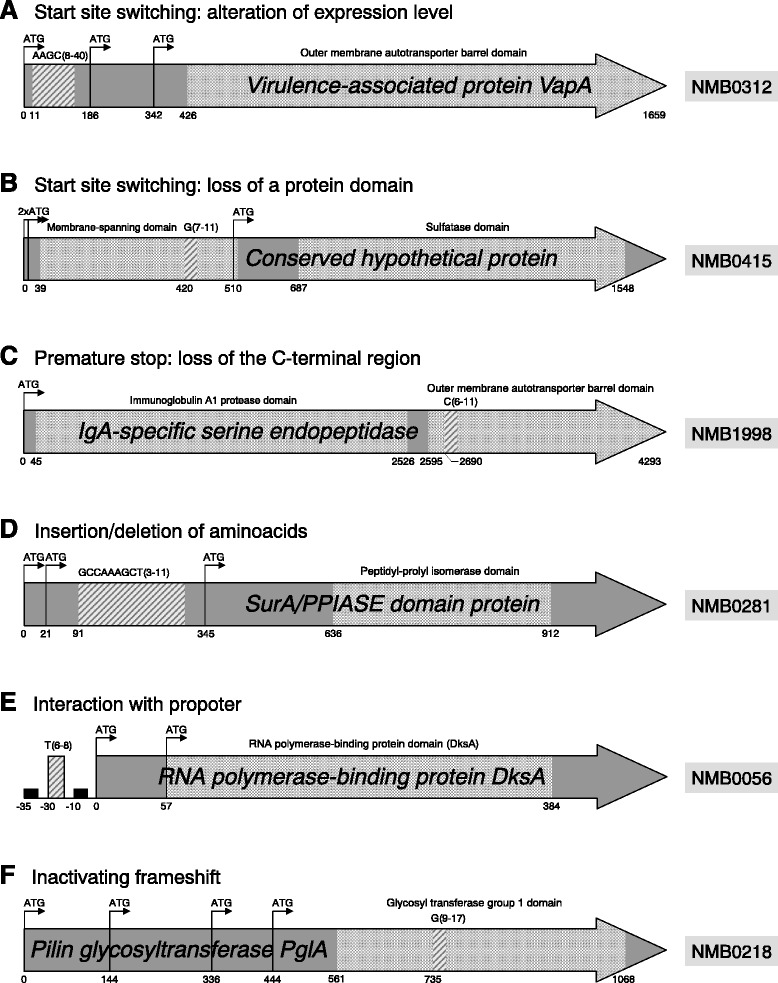
Schematic representation of five VNSSRs and their sequence context. **a** VNSSR causing translational start site switching. **b** VNSSR causing the loss of a membrane-spanning domain. **c** VNSSR leading to the loss of the peptide C-terminal region. **d** VNSSR introducing changes in the peptide sequence. E) VNSSR influencing the gene promoter. **f** VNSSR introducing an inactivating frameshift. Dark grey arrows represent open reading frames. Black arrows marked with ATG represent in-frame ATG translational start sites. Light grey boxes represent the annotated functional domains. Stripped boxes represent VNSSRs and the related tags indicate the repeat unit motif along with the minimum and maximum number of repetitions observed in the 20 analyzed genomes. Numbers below each gene indicate the position relative to the annotated translational tart site

We sought to gather new insights into the VNSSR-mediated regulatory mechanisms by analyzing the repeat sequence context in the 52 strong candidate phase variable genes. Nineteen of the 55 VNSSRs associated with strong candidate genes were surrounded by multiple in-frame 5′-ATG-3′ translational initiation codons (Additional file 5). These VNSSRs can shift the origin of translation between alternative start sites, resulting in the modulation of gene expression [11, 46]. Such modulation can arise either from the switching between start sites associated with different expression levels or from the production of different protein isoforms, as may be the case for the NMB0312 (Fig. 4a) and NMB0415 open reading frames (ORFs) (Fig. 4b), respectively. Different peptide isoforms may also result from VNSSRs located in the 3′ portion of the gene [47]. In this location, the VNSSR can bring the C-terminal peptide portion out of frame while still allowing the translation of a functional protein. Two ORFs, NMB1998 (Fig. 4c) and NMB0039, were consistent with this mechanism. In contrast, VNSSRs present within the NMB1969, NMB1818 and NMB0281 reading frames were either a 3- or a 9-nucleotides repeat. These VNSSRs do not interfere with the reading frame but can still influence the cell phenotype by producing proteins with different structures and/or post-translational profiles (Fig. 4d). Also, 15 of the 55 analyzed VNSSRs were predicted to interfere with the associated gene promoters, as represented by the T-homopolymeric tract located upstream of the NMB0056 start (Fig. 4e). The remaining 16 repeats were predicted to introduce frameshifts likely resulting in on-off type of transcriptional regulation, as proposed for NMB0218 (Fig. 4f).

### Most strong candidate phase variable genes are not specific to *N. meningitidis*

Among the 52 strong candidate phase variable genes, 6 were identified only within *Nm* through sequence database comparison. The GC content of these genes largely deviates from the typical meningococcal value (51-52 %), suggesting acquisition through lateral gene transfer from outside the genus. Conversely, orthologous copies of the remaining 46 genes were also identified in other *Neisseria* species (Additional file 2: Figure S9). Among these, 42 were associated with the same SSRs observed in *Nm*, indicating that these SSR-contingency loci arose before meningococcus speciation. In the remaining four cases (NMB0281, NMB0415, NMB1668 and GNMG2136_1693) the SSR associated with the gene was detected in *Nm* only, suggesting that these contingency loci have evolved following *Nm* speciation (Additional file 3).

Orthologous copies of 8 strong candidate phase variable genes were also identified in species outside of the *Neisseria* genus (Additional file 2: Figure S9), including *Cardiobacterium hominis*, *Haemophilus aegyptius*, *Haemophilus haemolyticus*, *Haemophilus influenzae*, *Kigella Dentrificans* and *Streptococcus pneumoniae*. Among these, the NMB1525 encoded gene identified in the three *Haemophilus* species and the 4 opacity proteins identified in *S. pneumoniae,* were associated with the same SSR as in *Nm* (NMB1636 example shown in Additional file 2: Figure S10), raising the hypothesis that SSR contingency loci are transferred across species through lateral DNA exchange.

### VNSSRs reorganization following *N. meningitidis* speciation primarily involved cell surface determinants

Among the 49 VNSSRs associated to the 46 strong candidate genes identified in multiple taxa, 22 (45 %) were found to be significantly longer (greater median number of motif repetitions; Wilcoxon test *p* ≤ 0.01) in *Nm* than in other species, while 3 were found to be significantly shorter (Additional file 3). Twelve and 3 of the repeats with increased length in *Nm* were G/C and A/T homopolymeric tracts, respectively, along with 7 other non-homopolymeric SSRs. These repeats were primarily associated with cell surface determinants, including surface receptors, surface transporters, pili and lipopolysaccharide biosynthetic enzymes. The 3 VNSSRs with a reduced size in *Nm* included a poly-T and a poly-C homopolymeric tracts, respectively associated with a hypothetical protein (NMB1209) and to a ferric enterobactin receptor (NMB1988), and a 5′-CTTCT-3′ repeat associated to an opacity protein (NMB0926). Also in this case, an association (2 genes out of 3) with surface exposed proteins was observed.

Overall, these results suggest that evasion of host immune responses, along with changes in surface adhesion properties and tissue tropism, have likely been the main drivers of SSR contingency loci evolution within *Nm*.

### VNSSRs chromosomic loci are fixed in the meningococcal population

Chromosomic loci containing the 324 identified VNSSRs were tested for signatures of selective pressures that could confirm a beneficial role (increased fitness) of those loci and justify their over-abundance in *Nm* species.

Only 14 (4.3 %) VNSSR loci, all associated with A/T homopolymeric tracts, showed a significant deviation from neutrality with all the applied tests (Additional file 6). Signatures of positive selection (negative Tajima and Fu-Li statistics) were identified in the upstream region of the two strong candidate phase variable genes *mtrF* (NMB1719), a surface-exposed efflux-pump component involved in hydrophobic antimicrobial resistance [48], and the transcription factor *dksA* (NMB0056). A signature of negative selection (positive Tajima and Fu-Li statistics) was detected in the upstream region of the moderate candidate phase variable gene *sstT* (NMB2133), which also encodes for a surface transporter. All other signals were identified in VNSSRs associated to weak candidate phase variable genes. The remaining 310 (95.7 %) tested loci did not show signatures of selection, supporting the hypothesis that SSR contingency loci have reached an equilibrium state and are now fixed in the population.

## Discussion

After the first *Nm* complete genome sequence became available [49], the highly abundant SSRs were largely associated with genes involved in host adaptation, commensalism and virulence. These initial studies on *Nm* SSRs contingency loci were based on analysis of individual genomes, strains MC58 [9] and Z2491 [20]. With the technology available at that time, the generation of new genomic sequences and the validation of the newly proposed phase-variable genes were laborious and time-consuming tasks. Despite the challenges, a few years after the first meningococcal genome sequence was revealed, a study reported a list of 69 *Nm* phase variable genes, 26 of which were experimentally confirmed [22].

Benefiting from the most recent sequencing technologies, the present study employed a 20 *Nm* genomes dataset, with a resolved phylogenetic structure [36], in a comparative genomic analysis aimed at the study of SSR-driven phase variation as a population-based, as opposed to single cell, adaptive strategy.

All analyzed strains were found to have more SSRs than expected in their chromosomes*.* A comparable number of SSRs were also found in two non-pathogenic strains, α14 and OX99-30304, questioning the hypothesis that SSRs-mediated phase variation is an adaptation associated with the evolution of virulence. Most of the identified SSRs were homopolymeric tracts, with an excess of A/T tandem repeats, suggesting slipped-strand mispairing as the primary mechanism driving the occurrence of SSRs. It has been proposed that slipped-strand mispairing is preferentially associated with homopolymeric tracts due to their high sequence redundancy [50] and favouring A/T repeats due to the lower stability of the DNA-DNA hybrid during bacterial genome replication [51].

The likelihood that repeat-associated genes undergo phase variation is variable. Efforts to identify molecular signatures indicative of such likelihood have defined a number of *cis* factors, including repeat sequence, size (number of motif repetitions) and sequence context, along with the associated gene function [9, 42]. Particularly, genes encoding for surface antigens were regarded as strong candidates for phase variation. We applied a more unsupervised approach, in which only the repeat sequence context and the distribution of its variation over the sampled phylogeny were considered. The over-representation of A/T repeats and their alleged higher propensity to cause transcriptional slippage compared to G/C repeats [51] would suggest these to be the main contributor of SSR mediated phase variation. Conversely, in agreement with previous findings [42], homopolymeric G/C repeats were the most frequent (50 % of cases) among the strong candidate genes pool, while weak candidate genes were almost exclusively associated with A/T repeats (95 % of cases). A primary involvement of C/G homopolymeric in the mediation of phase variation has also been described in *C. jejuni* [52]. This particular mutational pattern, together with the fact that we didn’t identify traces of selective pressure for most VNSSRs, suggest that there may be molecular drivers governing the mutability of these contingency loci. In support of this hypothesis, increased rates of phase variation have been associated with the loss of the *Dam* DNA methylase [53] and the two mismatch repair proteins *mutS* and *mutL* [54, 55].

Regarding non-homopolymeric repeats, 3-, 4-, 5-, 6- and 9-nucelotides VNSSRs were also found to be abundant. Three-, 6- and 9-nucleotides VNSSRs do not disrupt the reading frame and their abundance may be justified by a reduced selective pressure for their stability. Abundance of 5-nucleotide VNSSRs is due to the fact that 6 out of 8 cases are represented by the same 5′-CTTCT-3′ repeat associated to different paralogs of an opacity protein (NMB0442). Interestingly, a homolog of this opacity protein, coupled with the same SSR, was also identified in *Streptococcus pneumoniae* genome (Additional file 2: Figure S9), providing evidence that SSR-contingency loci can be transferred across different species, or strains of the same species and even different chromosomic loci of the same strain through lateral gene transfer. Conversely, abundance of 4-nucleotides VNSSRs, which has been reported both in *Nm* and in *Haemophilus influenzae* [56], has no obvious interpretation. Given the conservation of those repeat sequences and their proximity to the translational origin, the interaction with yet unidentified regulatory elements may be involved. One such regulatory mechanism has been described for the TAAA repeat associated with the *NadA* (NMB1994) promoter [35, 57].

The availability of high-throughput sequencing data enabled a high throughput experimental validation of *in silico* predicted SSR contingency loci. The rationale behind this approach was that DNA libraries used for Illumina sequencing were generated from a bacterial culture derived from a single colony. After an 18 h growth and approximately 36 bacterial replication cycles (assuming a growth rate of 2 duplications per hour), a library was expected to contain, and represent in the form of variable alleles, most of the VNSSRs variation. This test provided circumstantial evidence for phase variability of 93, 72 and 27 % of the strong, moderate and weak candidate genes respectively, leading to a total of 115 experimentally confirmed phase variable genes. This approach also allowed estimating VNSSRs variation frequencies. The 115 validated VNSSR showed an average proportion of alternative alleles of 0.71 %, indicating an average mutation rate of once every seven cell replication cycles. However, given the contribution of environmental, population and molecular factors to the mutability of SSRs in phase variable loci, it is likely that real frequencies may differ from those we observed in vitro.

SSR contingency loci were over-represented among genes related to cell surface functions and structure or involved in DNA metabolism, primarily DNA restriction and modification. These results, which are in agreement with previous findings [9], reflect a primary involvement of SSR-mediated phase variation in microbe-host interaction. Dis-regulation of those genes, in fact, alters *Nm* ability to adhere to and invade host cells, its ability to scavenge required nutrients and to evade host immune defenses by generating antigenic variants [58]. Additionally, putative phase variable genes were found in several other functional classes, indicating that SSR-mediated gene regulation interferes with a set of metabolic functions wider than previously hypothesized.

Our attempt to understand how VNSSRs have evolved in *Nm* revealed that most strong candidate phase variable genes (46/52) are widespread to the *Neisseria* genus and that about half of them have undergone size change after meningococcal speciation. This is suggestive of the fact that most SSR-associated contingency loci have developed prior to *Nm* speciation and have subsequently evolved, possibly as a consequence of the process of adaptation to the human host environment. In agreement with this is the fact that identified phase variable genes were enriched for cell surface determinants, suggesting that evasion of host immune responses and changes in surface adhesion properties and tissue tropism are the main drivers of SSR contingency loci evolution in *Nm*. Regarding the remaining 6 genes that are specific to *Nm*, they were characterized by a G/C content that deviates from that of meningococcus (data not shown), suggesting a possible acquisition through horizontal gene transfer. Phase variable genes candidates were also identified in species outside the *Neisseria* genus (Additional file 2: Figure S9). Interestingly, all of them are human pathogens that coexist in the respiratory flora of healthy individuals. Moreover, we found that genes shared by *Nm*, *S. pneumoniae* and three *Haemophilus* members were associated with the same repeat element as in *Nm*, supporting the possibility that SSR contingency loci are exchanged across species through lateral DNA exchange. Overall, our findings suggest that SSR contingency loci have been acquired by *Nm* either by spontaneous evolution, inheritance from an ancestral species or acquisition by means of lateral DNA exchange.

In the context of a commensal organism, constantly interacting with its host, the onset of new mutations or allele variants may lead to an increased fitness (mutations are favored in the population in a process called positive selection), a reduced fitness (mutations are selected against in a process called negative selection) or have no effect on fitness (mutations are neither selected for nor against and are said to be neutral) [59]. One of the most accredited hypotheses is that VNSSRs have evolved as an adaptive strategy, in organisms like *Nm*, to increase their fitness in the hostile and mutable host environment [7]. We tested all VNSSR-containing loci for signatures of selection that could confirm a beneficial role of those loci and justify their over-abundance. Surprisingly, the analysis revealed that most SSR-contingency loci are not under selective pressure, suggesting that *Nm* has had enough time to adapt to its niche and has apparently reached an equilibrium state. It must be noted, however, that substantial homologous recombination and repeated population expansions and bottlenecks, along with possible sampling biases, are likely to confound or even obscure neutrality tests results. Nonetheless, we envision that, applying this approach to a wider, unbiased strain collection, coming from a single outbreak, will have the potential of providing valuable insights into the understanding of VNSSRs evolutionary processes.

Despite a proportion of coding DNA in *Nm* higher than 80 % [20, 36, 49], we observed that 51 % of the identified VNSSRs are found within intergenic regions. This bias toward intergenic VNSSRs, which may be explained by a reduced selective pressure along non-coding regions, can also be indicative of a regulation of gene expression occurring at the transcriptional level. This type of VNSSRs can indeed modulate promoter efficiency by changing the relative spacing between key promoter elements [57]. Differently, intragenic VNSSRs, which can induce frameshift mutations, are responsible for an on/of type of regulation occurring at the translational level [13]. Additionally, given the existence of alternative translational start codons, intragenic VNSSRs located in the 5′ portion of a gene can switch between multiple active reading frames, with the potential of affecting the level of gene expression [11] or producing alternative peptide isoforms [46]. In the present analysis we observed that most intragenic VNSSRs are located in the proximity of the translational start site and that among the 52 strong candidate phase variable genes, there are 19 cases in which the VNSSR was found between two or more in-frame ATG translational start sites. Twenty other VNSSRs were predicted to either interact with the gene promoter or to produce alternative peptides isoforms. Overall, collected evidences suggest that SSR-contingency loci have evolved in such a way to allow the maximum degree of flexibility, reflected by their ability to finely modulate gene expression level, or the functionality of the resulting protein, rather than merely switching between the two extreme cases of a gene being expressed or silenced through the introduction of frameshifts.

## Conclusions

In conclusion, this analysis, which capitalized on the most recent DNA sequencing technologies, confirmed the fundamental contribution of SSR contingency loci in promoting phenotypic variation and their deep implications in *Nm* survival and adaptation to the surrounding environment. Our unsupervised approach allowed the generation of a panel of 277 genes whose expression may be controlled or influenced by SSR elements and to provide corroborative evidence for the phase variability for 115 of them. Functional characterization of these genes highlighted an enrichment for cell surface determinants, which included members of the adhesins, evasins, lipopolisaccharide biosynthesis and nutrient scavenging protein families. Such proteins have been attributed multiple roles in attachment of bacterial cells to host membranes [60, 61], in regulating bacterial resistance to both innate and adaptive immune system [62–64] and as major determinants of meningococcal invasive disease [33]. SSR-mediated phase variation has also been reported to control the expression of multiple immunogens currently included in commercial meningococcal vaccines [57, 65, 66] and to modulate resistance to antimicrobial agents [67–69]. We therefore envision that future studies on meningococcal microbe-host interaction and studies aimed at the identification of new vaccine antigens and antimicrobial molecules will benefit from this comprehensive characterization of the meningococcal putative phase variable genes repertoire.

## Methods

### Genomic dataset

This analysis was based on a dataset previously described [36]. The 20 genome sequences available were derived from isolates belonging to phylogenetic clades PC32/269 (MC58 [AE002098.2], H44/76 [CP002420], CU385 [AEQJ00000000], M04-240196 [CP002423], M01-240013 [AEQL00000000] and M13399 [AEQG00000000]), PC8/11 (G2136 [CP002419], 961-5945 [AEQK00000000], FAM18 [AM421808], M6190 [AEQF00000000] and ES14902 [AEQI00000000]) and PC41-44 (OX99-30304 [AEQE00000000], M0579 [AEQH00000000], NZ-05/33 [CP002424] and M01-240149 [CP002421]). Five other sequences were derived from isolates belonging to the following MLST groups: CC4281 (053442 [NC_010120.1]), CC53 (α14 [AM889136]), CC213 (M01-240355 [CP002422]), CC4 (Z2491 [AL157959]) and ST751 (N1568 [AEQD00000000]). Genome sequences are available online from the GenBank database through the reported accession numbers.

### SSRs identification, clustering and comparison

Tandemly repeated motifs, from 1 to 10 nucleotides in length, were identified in each genome using a Perl program developed *ad hoc*. Based on former repeat-associated phase variable genes investigations [9, 17], size cut-offs applied to the repeat search were as follows: 6 repetitions for homopolymeric tracts, 5 for 2-nucleotides, 4 for 3-nucleotides, 3 for 4-nucleotides, 4 for 5-nucleotides and 3 for 6- to 10-nucleotides repeats. The program allowed for the identification of imperfect simple sequence repeats (parameters applied are reported and described in Additional file 7).

In order to establish clusters of orthologous SSRs, all the identified repeats, along with their 50-nucleotides 5′ and 3′ flanking regions, were annotated as features on the respective genome sequences following the GenBank flat file format. All genomes were aligned with the ProgressiveMauve multiple alignment algorithm implemented in the MAUVE v2.3.1 toolkit using the seed-family option [70]. SSRs present in syntenic chromosomic regions, sharing the same repeat sequence and a minimum of 70 % sequence identity over 60 % coverage were identified as orthologous features.

For each cluster of orthologous SSRs, SSR sequences, along with their flanking 50 nucleotides, were aligned using the MUSCLE aligner [71]. Each alignment was then analyzed in order to identify SSR length polymorphisms across different genomes. This resulted in 492 clusters of orthologous SSRs showing evidence of interstrain length polymorphisms. For each variable number simple sequence repeat (VNSSR) the degree of size heterogeneity was assessed by the Nei’s diversity index [DI = 1-∑(allele frequency)^2^] [41].

### Putative phase variable genes identification

Identity, distance and orientation of genes containing, or located next to VNSSRs were extracted using a Perl program developed *ad hoc*. One hundred and fifty-eight (158) VNSSRs, located within 200 nucleotides preceding the associated genes translational start site, and 166 VNSSRs, located within the genes coding region, were selected for further analyses.

Classification into weak, moderate and strong candidate phase variable genes was based on three parameters: *i*) degree of SSRs overrepresentation compared to a neutral expectation [calculated using a previously described approach [72]], *ii*) the range of VNSSR length variation (difference between the longest and the shortest repeat across multiple genomes) and *iii*) the distribution of the variation over the meningococcal phylogeny, as defined in [36]. Homopolymeric VNSSRs with a length variation range of a single unit motif and not being over-represented (<9 repetitions for A/T tracts and <7 repetitions for G/C tracts) were classified as weak candidates. VNSSRs characterized by a length variation range greater than one repeat unit and showing length variation among members of the same clonal complex in at least two clonal complexes were classified as strong candidates. Remaining VNSSRs were classified as moderate candidates.

The sequence context of the 55 VNSSRs associated with the 52 strong candidate genes was further analysed in order to elucidate possible implications of VNSSRs variation on the associated gene expression. The relative position of the repeats, in-frame ATG translational start-sites and annotated peptide functional domains were extracted (Additional file 5) and manually inspected. The identity and location of peptides functional domains were predicted by HMM motif searches on Pfam [73] and TIGRfam [74] databases.

### Identification of functional roles associated to SSR contingency loci

TIGR main functional roles [45] associated with meningococcal putative phase variable genes were tested for enrichment using the chi-squared test followed by Bonferroni correction for multiple testing. Functional categories associated with a corrected *p* value ≤ 0.01 were assumed to be enriched for VNSSR-associated genes.

### Illumina sequencing and data analysis

Genomic sequences of strains G2136, M01-240355, M04-240196, MC58 and NZ-05/33 were re-sequenced using the Illumina HiSeq2000 platform. For each strain, the stocked inoculum was streaked onto agar-chocolate plates and grown overnight (18 h) at 37°. About 30 colonies were harvested into phosphate buffered saline and further processed for phenol-chloroform DNA extraction. Purified DNA was fragmented using the Covaris M220 Focused-ultrasonicator™ and further processed using the Beckman Coulter SPRI-TE™ instrument following the manufacturer’s instructions. Clusters of flowcells were generated via the Illumina cBot cluster amplification system and the TruSeq PE Cluster Kit (v.2). Sequencing was carried out on an Illumina HighSeq2000 sequencer following manufacturer guidelines [75].

The 66 nucleotide paired-reads coming from the sequencer were mapped over the corresponding reference genomes using the BWA toolkit v0.5.9 [76] with parameters q = 30 (quality cut-off for read trimming), e = 5 (maximum number of gap extensions), O = 8 (gap-open penalty) and E = 3 (gap-extension penalty). Duplicate reads were filtered out using Picard v1.58. Using this approach it was possible to detect length polymorphisms of SSRs with motifs up to 5-nucleotides long. Reads mapping over the chromosomic regions containing the predicted VNSSRs, spanning the entire repetitive sequence plus at least 4 flanking nucleotides on both 5′ and 3′ extremities, and having a Phred-scaled MAPping quality ≥30 were extracted for each VNSSR and analyzed for SSR length polymorphisms. SSRs showing a proportion of alternative alleles >0.002 were considered to be variable. Due to limitations of the reads mapping procedure, it was not possible to test repeats with a unit motif longer than 5 nucleotides or repeats found inside paralogous chromosomic regions. Finally, it was not possible to test those repeats whose overall length cannot be entirely covered by a single read.

Possible contribution of sequencing errors to the detection of false positive VNSSRs was evaluated by applying the same procedure to 100 randomly selected SSRs that showed no length polymorphisms in the 20-genome comparative analysis.

The regression analysis for new validated phase variable genes with an increasing number of tested genomes was performed as previously described [44]: the Heaps’ power law function *n = kN* ^*γ*^ was fitted to the data (for *N* > 1) with a least squares regression, where *n* is the number of validated phase variable genes, *N* is the number of genomes and *k* and *γ* are free parameters.

### Identification of putative phase variable genes across species

In order to assess whether the 52 putative phase variable genes were present in species other than *Nm*, their DNA sequence was searched against the NCBI Reference Sequence [77] and the Whole Genome Shotgun [78] databases using BLAST (BLASTN v.2.2) [79]. Matches with at least 80 % sequence identity over 50 % coverage of the query were extracted and further analyzed.

A similar approach was also used for the analysis of the evolution of VNSSRs associated to strong candidate genes. Orthologous VNSSRs present in different species were extracted and compared through the Wilcoxon test. VNSSRs having a greater or smaller median size (number of motif repetitions) and a difference-associated *p* value ≤ 0.01 were assumed to have grown or shrunk in meningococcus, respectively.

### Neutrality tests on VNSSR-containing loci

Each cluster of orthologous VNSSRs was extracted, along with their flanking 50 nucleotides, and aligned using the MUSCLE aligner [71]. Each alignment was subsequently tested for signatures of selective pressure using the VariScan software package [80] with the following parameters: StartPos = 1, EndPos = 0, RefPos = 1, Outgroup = none, BlockDataFile = none, RefSeq = 1, RunMode = 12, UseMuts = 1, FixNum = 0, NumNuc = 4 and SlidingWindow = 0. This configuration analyzes the entire alignment region and computes the Tajima D, Fu-Li D* and Fu-Li F* neutrality tests statistics [81, 82]. VNSSRs loci showing deviation from neutral evolution (*p* ≤ 0.05) with all 3 tests were considered to be under selective pressure.

## Additional files

Additional file 1: Number of SSRs identified in the 20 Neisseria meningitidis genomes collection. (XLSX 37 kb)
Additional file 2: Figures S1-S10. with their legends. (PDF 500 kb)
Additional file 3: List of putative VNSSRs identified in the 20 *Neisseria meningitidis* genomes collection. (XLSX 48 kb)
Additional file 4: VNSSRs validation by Illumina sequencing. (XLSX 108 kb)
Additional file 5: Sequence context of the 55 VNSSRs associated to strong candidate genes. (XLSX 58 kb)
Additional file 6: Neutrality test results for the 324 VNSSRs-containing loci. (XLSX 63 kb)
Additional file 7: Parameters applied for SSR identification. (XLSX 32 kb)

## Abbreviations

A/T: Adenine or thymine nucleotide
BLAST: Basic local alignment search tool
CC: Clonal complex
DI: Nei’s diversity index
G/C: Cytosine or guanine nucleotide
HMM: Hidden Markov model
MLST: Multilocus sequence typing
*Nm*: *Neisseria meningitidis*
ORF: Open reading frame
SSR: Simple sequence repeat
TIGR: The Institute for Genomic Research
VNSSR: Variable number simple sequence repeats
