# Supplementary material for: In-silico prediction and deep-DNA sequencing validation indicate phase variation in 115 Neisseria meningitidis genes
Source: BMC Genomics. 2016 Oct 28;17:843. doi: 10.1186/s12864-016-3185-1 (PMC5084427; doi:10.1186/s12864-016-3185-1)
Supplement: Additional file 2: Figures S1-S10. — with their legends. (PDF 500 kb) [file 12864_2016_3185_MOESM2_ESM.pdf]

Figure S1. Number of SSRs identified in the 20 analyzed *Nm* genomes (*N. meningitidis* real) and in a collection of 20 genomes generated *in silico* (*N. meningitidis* simulated). The 20 simulated genomes were generated such that they had same length (2272360 bp), same codon frequencies for coding regions and same GC content for the non-coding regions (53.08%) as the MC58 genome. Thick horizontal lines and box boundaries represent median and central interquartile range, respectively. Box whiskers extend to the data extremes. "All SSRs": data relative to all SSR types pooled together.

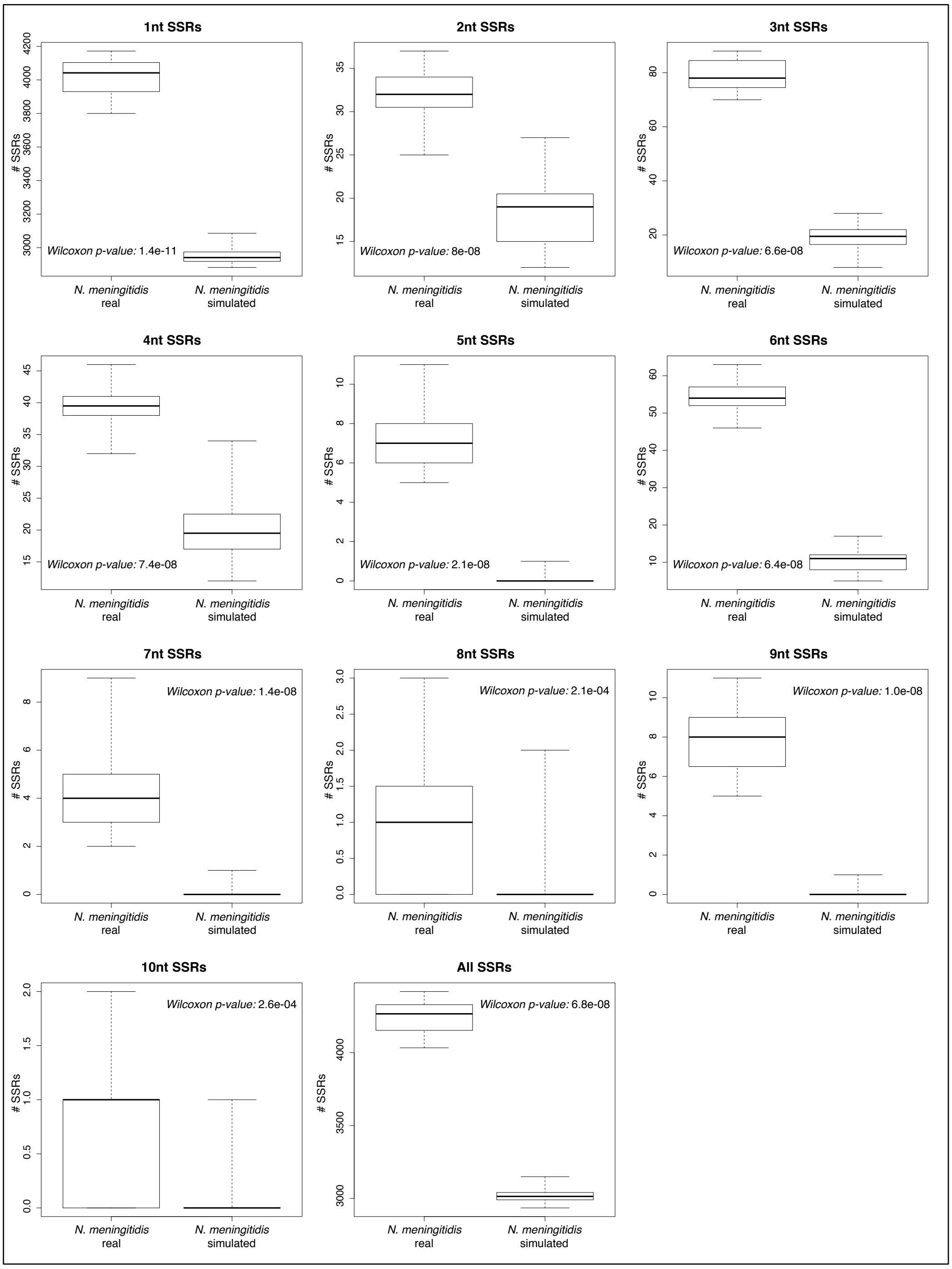

Figure S2. Correlation between VNSSRs size (number of unit motif repetitions) and variability (Nei's diversity index). A) VNSSRs associated to strong candidates genes. B) VNSSRs associated to moderate candidate genes. C) VNSSRs associated to weak candidate genes. X-axes represent the number of times the VNSSR unit motif is repeated. Y-axes represent the VNSSRs associated Nei's diversity indexes. '○': homopolymeric VNSSRs. '×': all non homopolymeric VNSSRs. Solid lines represent the least squares fit of the homopolymeric VNSSRs. Dashed lines represent the least squares fit of the non-homopolymeric VNSSRs. 'Slope': slope of the line defined by the least squares fit. 'R': Pearson's correlation coefficient and associated *p* value.

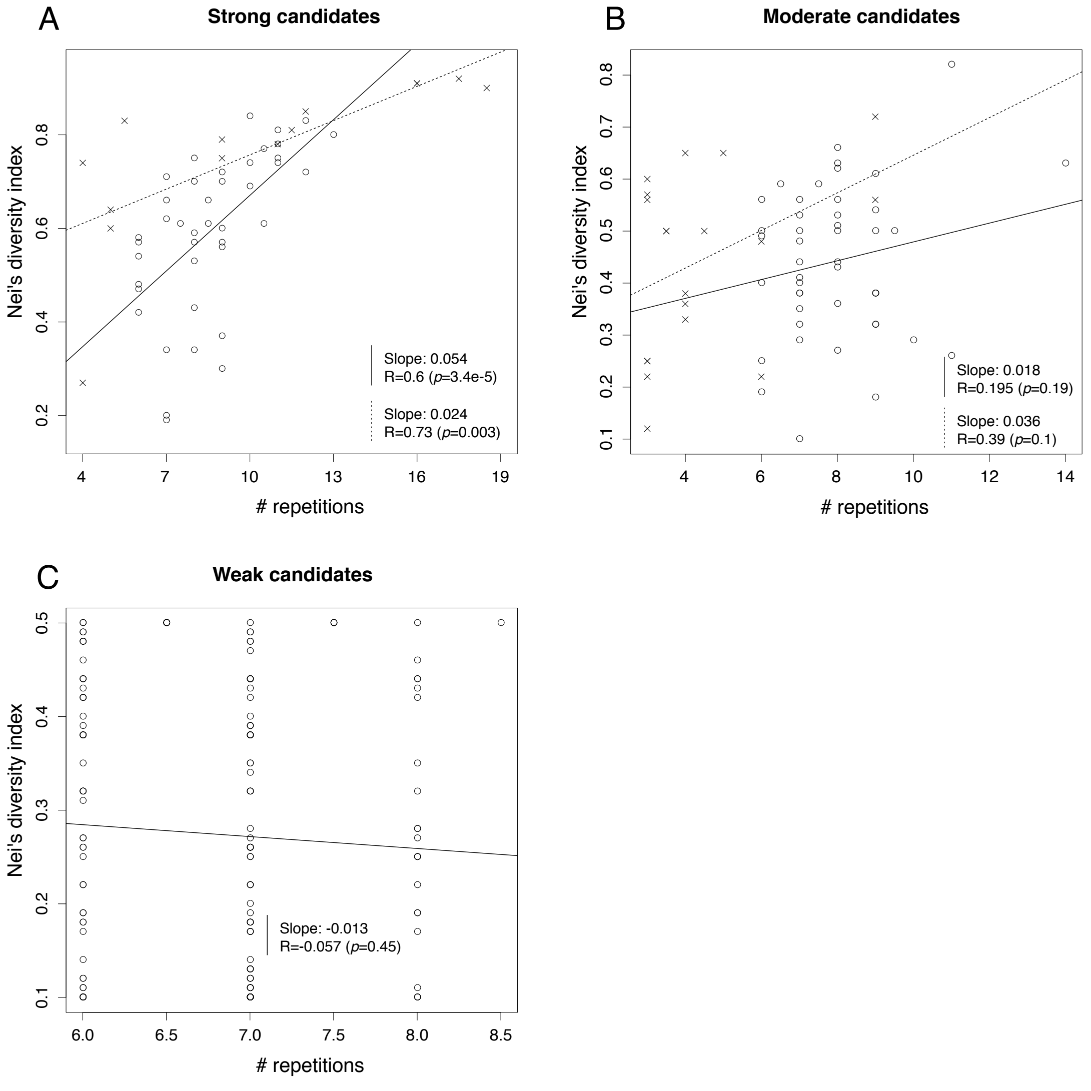

Figure S3: Proportion of sequencing reads showing polymorphic SSR variants. Sample: data relative to the 324 predicted VNSSRs. Neg. control: data relative to 100 SSRs randomly-selected among those that were predicted as non-variable by the comparative genomic analysis. Thick horizontal lines and box boundaries represent median and central interquartile range, respectively. Box whiskers extend up to 1.5 times the interquartile range.

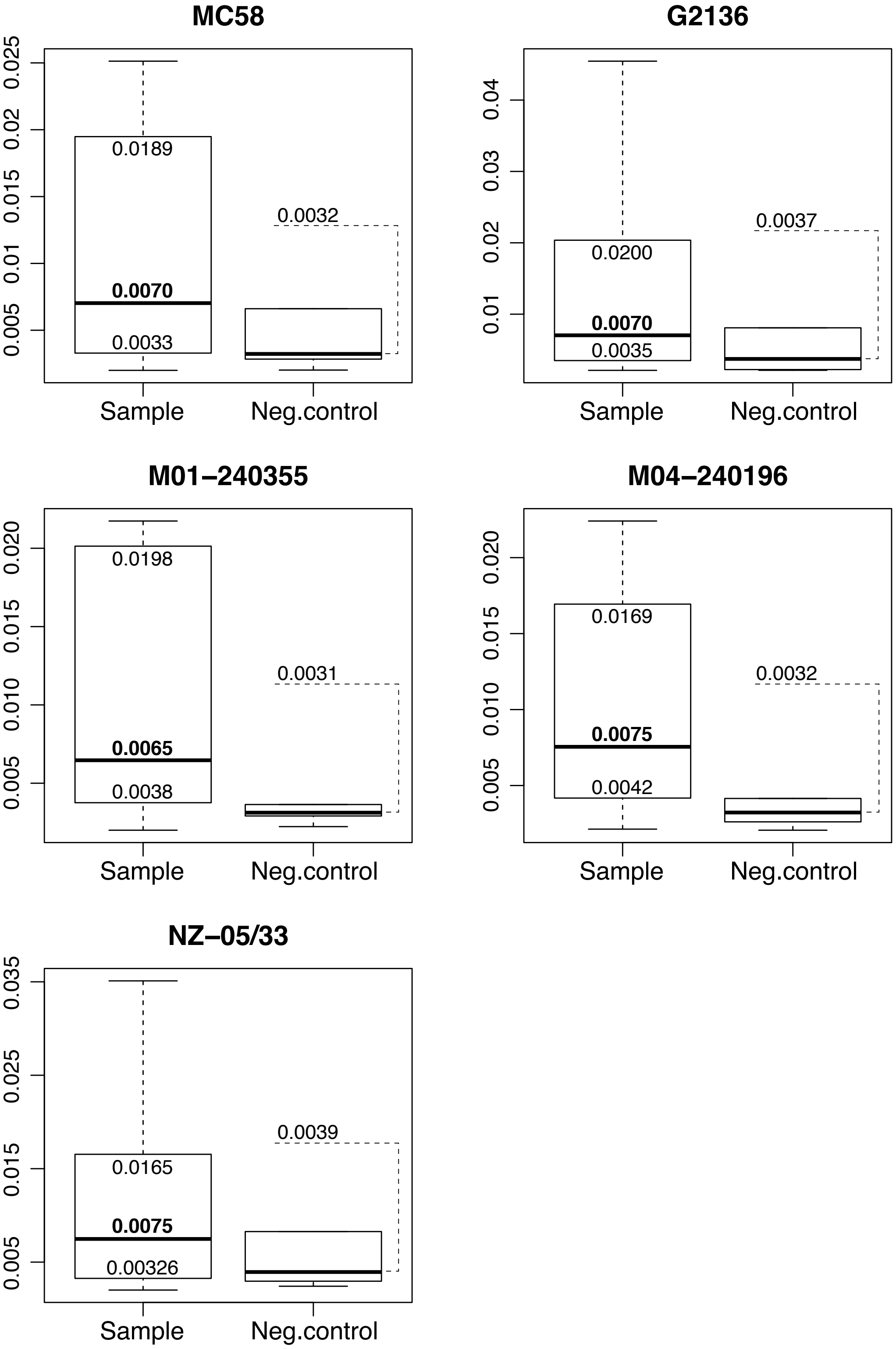

Figure S4: A) Number of genomes in which the 70 core VNSSRs were confirmed by deep sequencing data. B) Homopolymeric VNSSRs size represented as a function of the number of genomes in which they were confirmed by deep sequencing data. Dotted line represents the size threshold for which most homopolymeric VNSSRs confirmed in 1, 2 or 3 genomes were below the threshold and all homopolymeric VNSSRs confirmed in 4 or 5 genomes were equal or above the threshold (chi-square p = 1e-11).

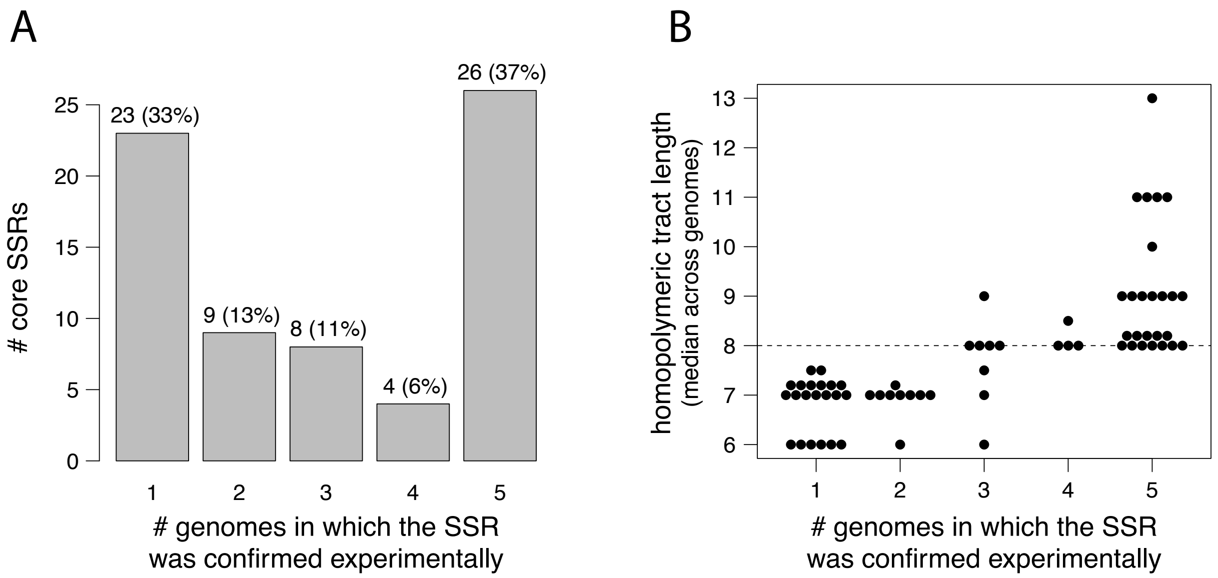

Figure S5: Correlation between the proportion of polymorphic reads, derived from the deep sequencing data, and the Nei's diversity index, derived from the 20 genomes comparative analysis. A) Least squares regression. B) Chi-square statistics comparing the proportion of VNSSRs showing a proportion of polymorphic reads < or ≥ the average value (-2.28) with the proportion of VNSSRs associated with a Nei's diversity index < or ≥ 0.5 (central value). Only data relative to the 70 core VNSSRs validated by deep sequencing are shown.

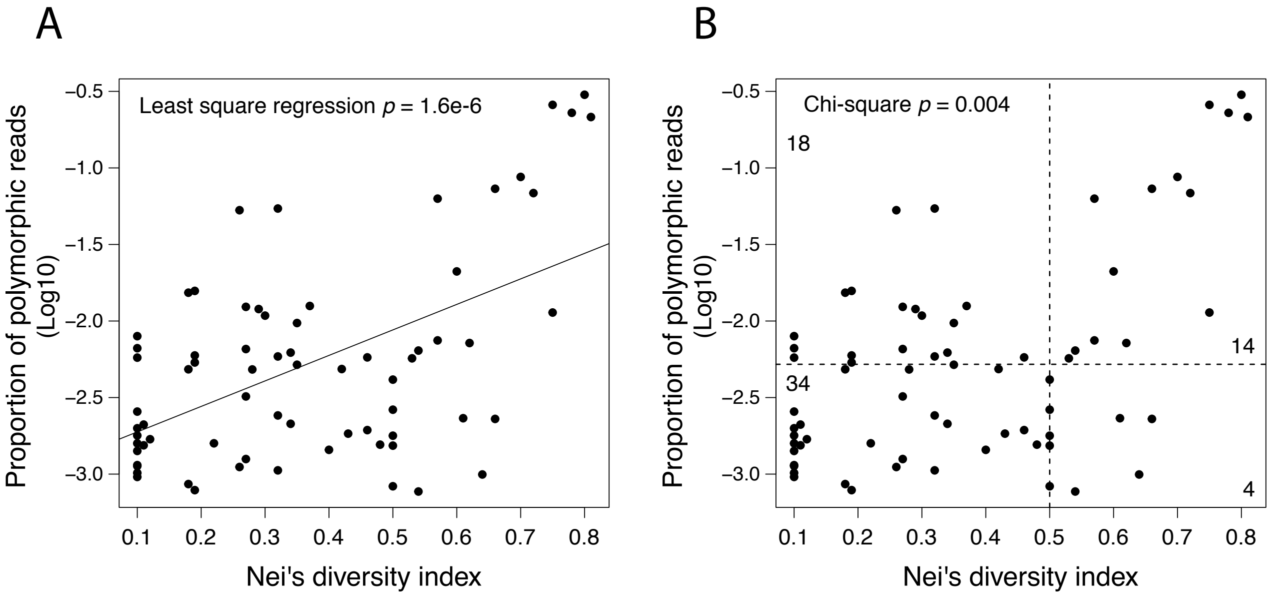

Figure S6: Number of confirmed phase variable genes plotted as a function of the number of genomes available for deep sequencing. Least squares regression of the Heaps' law function to the data describing the number of newly discovered phase variable genes as a function of the number genome sequences. Black circles indicate the median of the distributions, while whiskers extend to data extremes. Heaps' law: $n=kN^{\gamma}$; where n represents the number of confirmed phase variable genes, N represents the number of genomes and k and γ are free parameters. Bracketed numbers indicate standard deviations.

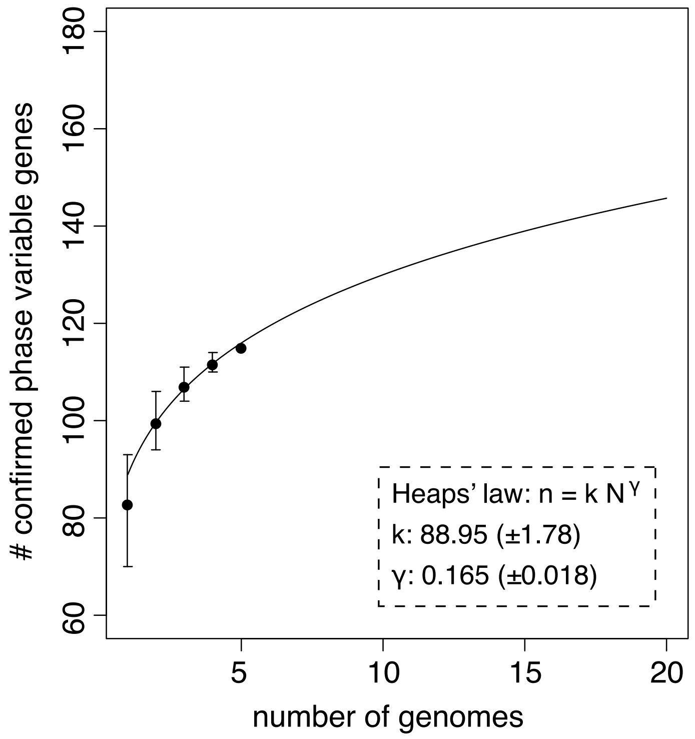

Figure S7: Comparison of present results with previously known or proposed phase variable genes. Experimental: 26 genes experimentally proofed to be phase variable. Strong: 19 genes that have been proposed as strong candidates for phase variation. Moderate/weak: 24 genes that have been proposed either as moderate or weak candidates. New: 230 genes not previously described to be associated with SSR-mediated regulation. Black, dark grey and light grey bars represent putative phase variable genes that we classified as strong, moderate and weak candidates for phase variation, respectively. White bars represent genes previously proposed as phase-variable that the present analysis failed to confirm.

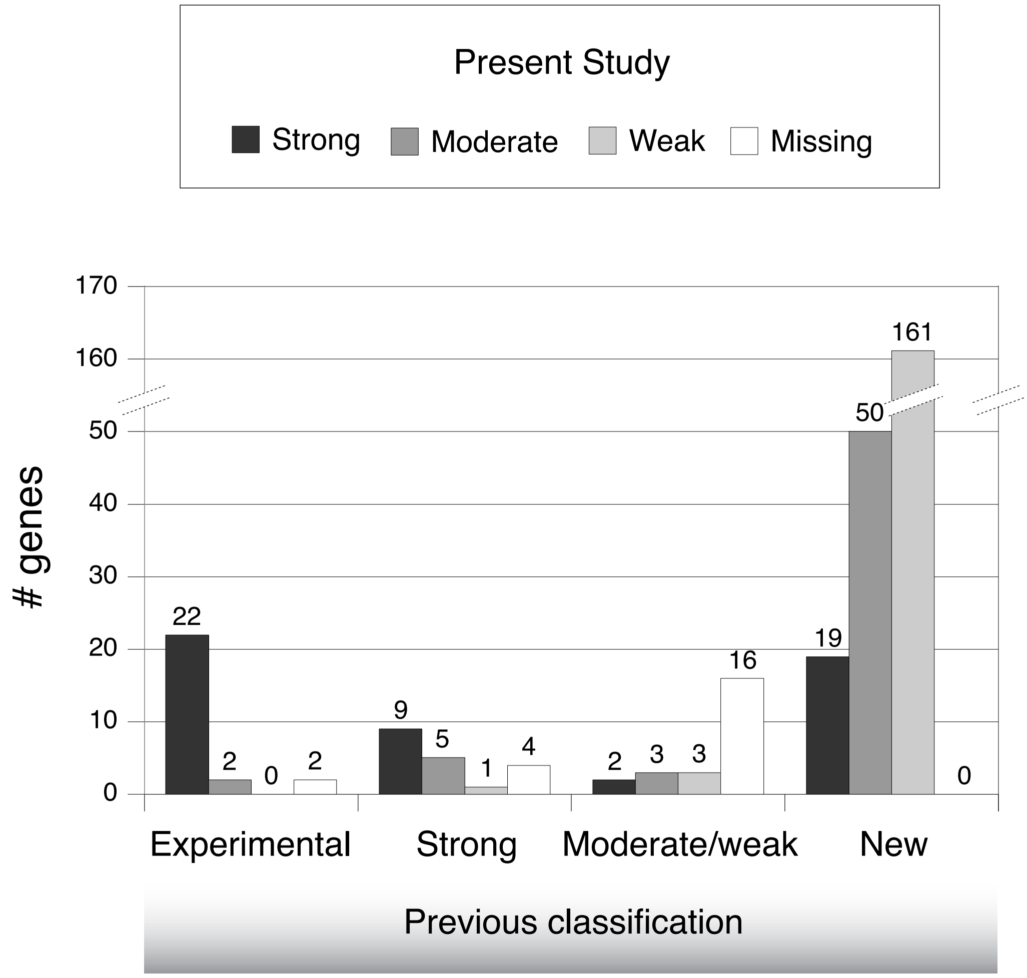

Figure S8: TIGR main functional roles represented by the 165 weak candidate phase variable genes. *X*-axis represents the proportion of putative phase variable genes present in each category. Numbers in brackets represent the number of putative phase variable genes over the total number of genes that are associated to a specific function. Genes associated with intragenic and intergenic SSRs are represented in dark and light grey, respectively. '*': Over-represented functional roles (Bonferroni adjusted p value ≤ 0.01). '+': Functional roles not previously described to be associated with SSR-contingency loci in *Nm*.

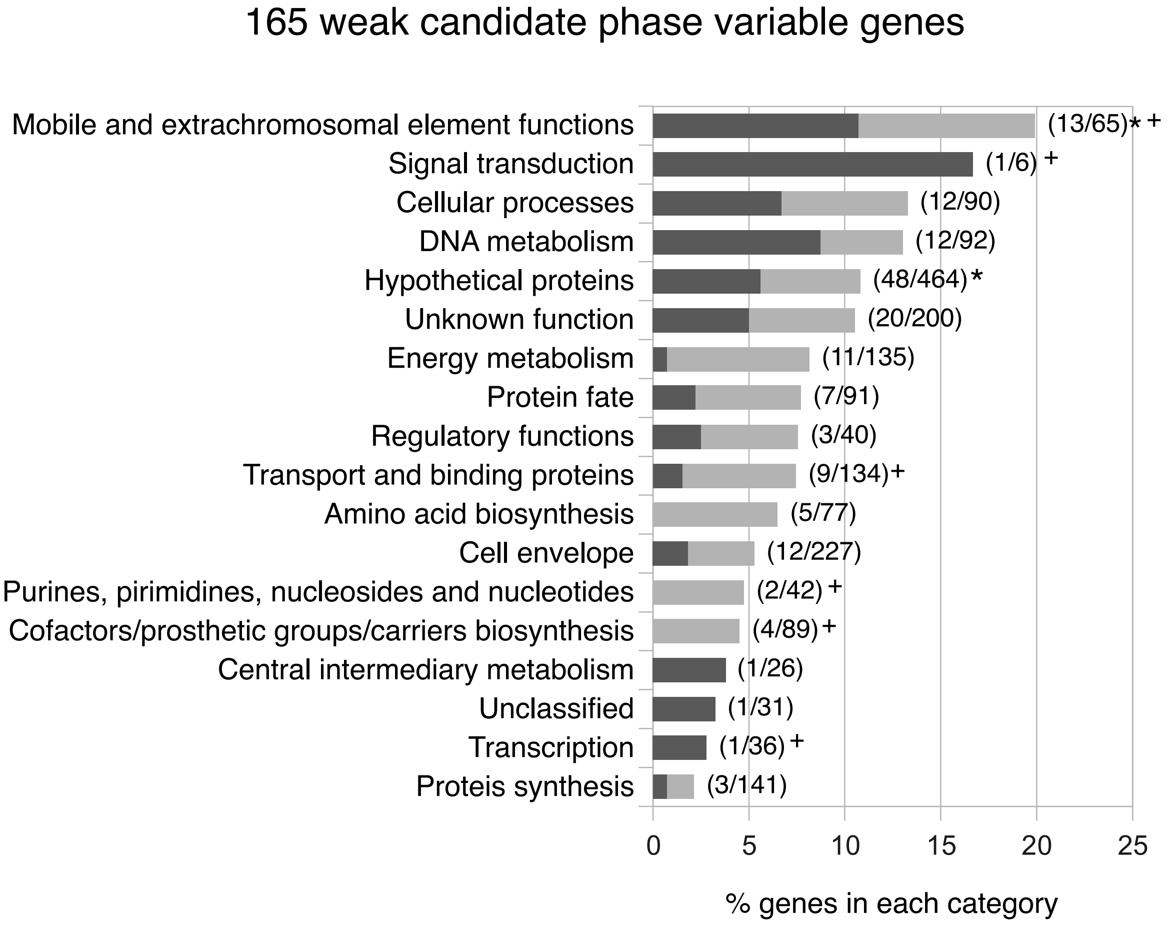

Figure S9: Strong candidate phase variable genes occurrence across bacterial species.

Taxonomic representation (showed as a collapsed cladogram) of bacterial species in which the 52 strong candidate phase variable genes have been identified. Colored text indicates species names, black text indicates organisms' family name (genus name in the case of "Neisseria") while grey text indicates organisms taxonomic class name. The red colored internal node represents the root. '*': Number of genes present in each species subset. Identified genes are listed inside the dashed line enclosed areas.

Figure S9

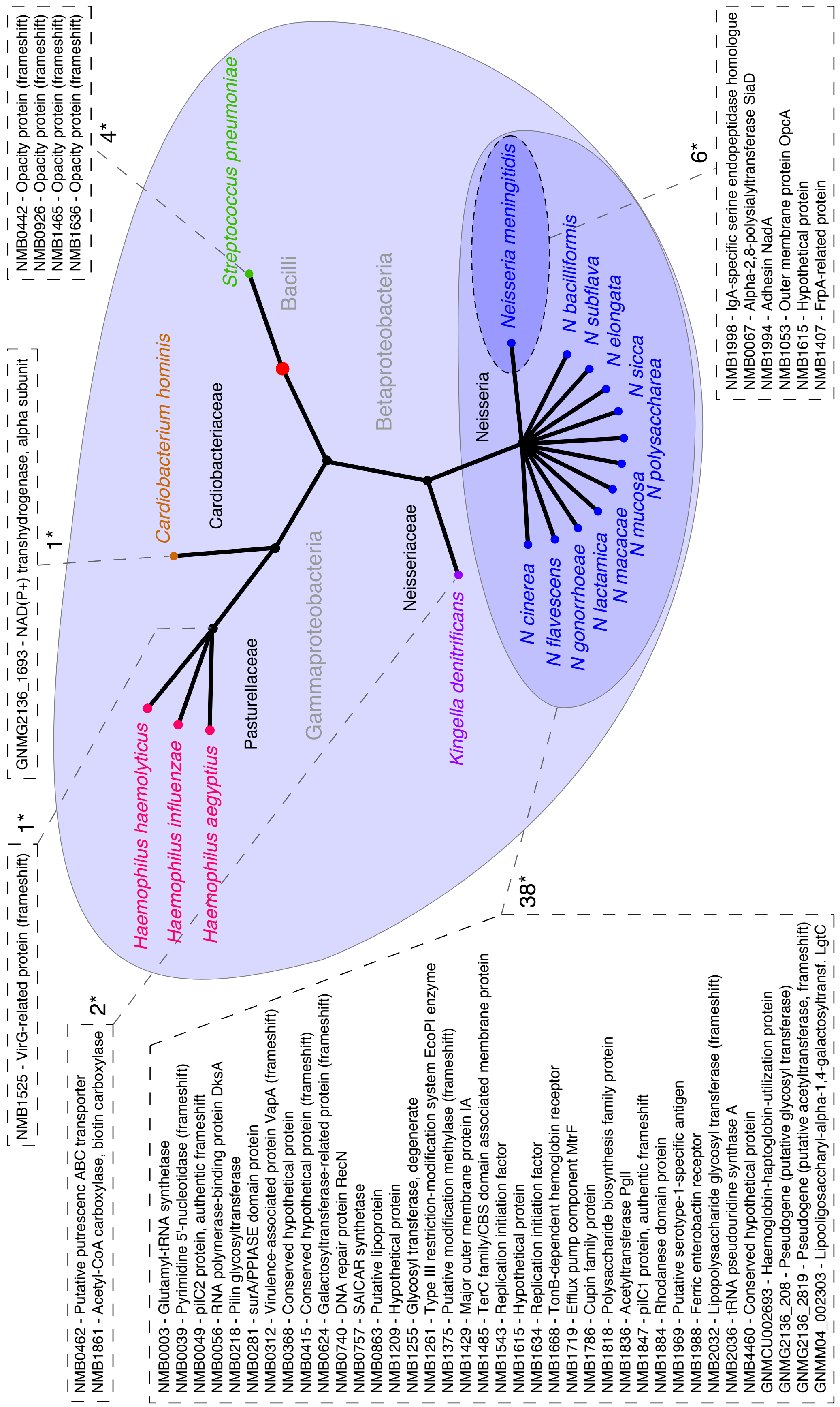
NMB1525 - VirG-related protein (frameshift)
1*
GNMG2136_1693 - NAD(P+) transhydrogenase, alpha subunit
1*
NMB0442 - Opacity protein (frameshift)
NMB0926 - Opacity protein (frameshift)
NMB1465 - Opacity protein (frameshift)
NMB1636 - Opacity protein (frameshift)
4*
Haemophilus haemolyticus
Haemophilus influenzae
Haemophilus aegyptius
Pasturellaceae
Cardiobacterium hominis
Cardiobacteriaceae
Streptococcus pneumoniae
Bacilli
Gammaproteobacteria
Betaproteobacteria
Neisseriaceae
Kingella denitrificans
Neisseria
Neisseria meningitidis
N bacilliformis
N subflava
N elongata
N sicca
N polysaccharea
N mucosa
N macacae
N lactamica
N gonorrhoeae
N flavescens
N cinerea
2*
NMB0462 - Putative putrescenc ABC transporter
NMB1861 - Acetyl-CoA carboxylase, biotin carboxylase
38*
NMB0003 - Glutamyl-tRNA synthetase
NMB0039 - Pyrimidine 5'-nucleotidase (frameshift)
NMB0049 - pilC2 protein, authentic frameshift
NMB0056 - RNA polymerase-binding protein DksA
NMB0218 - Pilin glycosyltransferase
NMB0281 - surA/PPIASE domain protein
NMB0312 - Virulence-associated protein VapA (frameshift)
NMB0368 - Conserved hypothetical protein
NMB0415 - Conserved hypothetical protein (frameshift)
NMB0624 - Galactosyltransferase-related protein (frameshift)
NMB0740 - DNA repair protein RecN
NMB0757 - SAICAR synthetase
NMB0863 - Putative lipoprotein
NMB1209 - Hypothetical protein
NMB1255 - Glycosyl transferase, degenerate
NMB1261 - Type III restriction-modification system EcoPI enzyme
NMB1375 - Putative modification methylase (frameshift)
NMB1429 - Major outer membrane protein IA
NMB1485 - TerC family/CBS domain associated membrane protein
NMB1543 - Replication initiation factor
NMB1615 - Hypothetical protein
NMB1634 - Replication initiation factor
NMB1668 - TonB-dependent hemoglobin receptor
NMB1719 - Efflux pump component MtrF
NMB1786 - Cupin family protein
NMB1818 - Polysaccharide biosynthesis family protein
NMB1836 - Acetyltransferase PglI
NMB1847 - pilC1 protein, authentic frameshift
NMB1884 - Rhodanese domain protein
NMB1969 - Putative serotype-1-specific antigen
NMB1988 - Ferric enterobactin receptor
NMB2032 - Lipopolysaccharide glycosyl transferase (frameshift)
NMB2036 - tRNA pseudouridine synthase A
NMB4460 - Conserved hypothetical protein
GNMCU002693 - Haemoglobin-haptoglobin-utilization protein
GNMG2136_208 - Pseudogene (putative glycosyl transferase)
GNMG2136_2819 - Pseudogene (putative acetyltransferase, frameshift)
GNMM04_002303 - Lipooligosaccharyl-alpha-1,4-galactosyltransf. LgtC
6*
NMB1998 - IgA-specific serine endopeptidase homologue
NMB0067 - Alpha-2,8-polysialyltransferase SiaD
NMB1994 - Adhesin NadA
NMB1053 - Outer membrane protein OpcA
NMB1615 - Hypothetical protein
NMB1407 - FrpA-related protein

Figure S10: Multiple sequence alignment of the Opacity protein encoded by the NMB1636 open reading frame. The sequence corresponding to the VNSSR is underlined. Bold characters represent the translational start (atg) and stop (tga) codons.

```
                          1  -->  10        20        30        40        50        60
N.meningitidis MC58    aaaatgaatccagcccccaaaaaaccttctcttctcttctcttctcttctcttctcttct
N.meningitidis Z2491   aaaatgaatccagcccccaaaaaac--------------------cttctcttctcttct
N.meningitidis FAM18   aaaatgaatccagcccccaaaaaac---------------cttctcttctcttctcttct
N.gonorrhoeae FA1090   aaaatgaatccagcccgcaaaaaaccttctcttctcttctcttctcttctcttctcttct
S.pneumoniae MLV-016   ................. no sequence information ..................

                      61        70        80        90       100       110       120
N.meningitidis MC58    cttctcttctcttctcttctcttccgcagcgcaggcggcaagtgaagacggcagccgcag
N.meningitidis Z2491   cttctcttctcttctcttctcttccgcagcgcaggcggcaagtgaagacggcagccgcag
N.meningitidis FAM18   cttctcttctcttctcttctcttccgcagcgcaggcggcaagtgaagacagcgggcacgg
N.gonorrhoeae FA1090   cttctcttctcttctcttctcttccgcagcgcaggcggcaagtgaaggcaatggccgcgg
S.pneumoniae MLV-016   cttctcttctcttctcttctcttccgcagcgcaggcggcaagtgaagacggcagtcgcag

                     121       130       140       150       160       170       180
N.meningitidis MC58    cccgtattatgtgcaggcggatttagcttatgccgccgaacgtattacccacgattatcc
N.meningitidis Z2491   cccgtattatgtgcaggcggatttagcctacgccgccgaacgcattacccacgattatcc
N.meningitidis FAM18   cccgtattatgtgcaggcggatttagcttatgccgccgaacgtattacccacgattatcc
N.gonorrhoeae FA1090   cccg---tatgtgcaggcggatttagcctacgccgccgaacgcattacccacgattatcc
S.pneumoniae MLV-016   cccgtattatgtgcaggcggatttagcctacgccgccgaacgcattacccacgattatcc

                                  sequence removed ...

                     781       790       800       810       820       830       840
N.meningitidis MC58    gggacgcttggaaaacacccgcttcaaaacccacgaagtctcattgggcatgcgctacca
N.meningitidis Z2491   gggacgcttggaaaacacccgcttcaaaacccacgaagtctcattgggcatgcgctacca
N.meningitidis FAM18   gggacgcttggaaaacacccgcttcaaaacccacgaagtctcattgggcatgcgctaccg
N.gonorrhoeae FA1090   gggacgcctggaaaacacccgcttcaaaacccacgaagcctcattgggcatgcgctaccg
S.pneumoniae MLV-016   gggacgcttggaaaacacccgcttcaaaacccacgaagtctcattgggcatgcgctacca

                     841   --|
N.meningitidis MC58    cttctga
N.meningitidis Z2491   cttctga
N.meningitidis FAM18   cttctga
N.gonorrhoeae FA1090   cttctga
S.pneumoniae MLV-016   cttctga
```
